# Fine Regulation of MicroRNAs in Gene Regulatory Networks and Pathophysiology

**DOI:** 10.3390/ijms26072861

**Published:** 2025-03-21

**Authors:** Mayu Seida, Koichi Ogami, Seiko Yoshino, Hiroshi I. Suzuki

**Affiliations:** 1 Division of Molecular Oncology, Center for Neurological Diseases and Cancer, Nagoya University Graduate School of Medicine, Nagoya 466-8550, Japan; 2 Institute for Glyco-core Research (iGCORE), Nagoya University, Nagoya 464-8601, Japan; 3 Center for One Medicine Innovative Translational Research (COMIT), Nagoya University, Nagoya 464-8601, Japan; 4 Inamori Research Institute for Science (InaRIS), Kyoto 600-8411, Japan

**Keywords:** miRNA, miRNA biogenesis, co-targeting, AGO syndrome, super-enhancer, miRNA 3′ tailing and 3′ trimming, TDMD, miRNA evolution, neuronal development

## Abstract

MicroRNAs (miRNAs) are ~22-nucleotide small non-coding RNAs that play critical roles in gene regulation. The discovery of miRNAs in *Caenorhabditis elegans* in 1993 by the research groups of Victor Ambros and Gary Ruvkun opened a new era in RNA research. Typically, miRNAs act as negative regulators of gene expression by binding to complementary sequences within the 3′ untranslated regions of their target mRNAs. This interaction results in translational repression and/or target destabilization. The expression levels and activities of miRNAs are fine-tuned by multiple factors, including the miRNA biogenesis pathway, variability in target recognition, super-enhancers, post-transcriptional modifications, and target-directed miRNA degradation. Together, these factors form complex mechanisms that govern gene regulation and underlie several pathological conditions, including Argonaute syndrome, genetic diseases driven by super-enhancer-associated miRNAs, and miRNA-deadenylation-associated bone marrow failure syndromes. In addition, as miRNA genes have evolved rapidly in vertebrates, miRNA regulation in the brain is characterized by several unique features. In this review, we summarize recent insights into the role of miRNAs in human diseases, focusing on miRNA biogenesis; regulatory mechanisms, such as super-enhancers; and the impact of post-transcriptional modifications. By exploring these mechanisms, we highlight the intricate and multifaceted roles of miRNAs in health and disease.

## 1. Introduction

In 1993, Victor Ambros and Gary Ruvkun’s research groups first reported the existence of a novel class of small non-coding RNAs, which were later named microRNAs (miRNAs) [1,2]. They demonstrated that the first miRNA they identified, lin-4, plays a crucial role in regulating postembryonic development in *Caenorhabditis elegans*. This was followed by the characterization of several heterochronic genes required to control developmental timing, including *lin-4*, *lin-14*, *lin-28*, and *lin-29* [3,4]. Ambros’s group isolated *lin-4* by combining restriction fragment length polymorphism (RFLP) mapping, transformation rescue, and open reading frame (ORF) prediction. By doing so, they found that *lin-4* does not encode a protein but rather a small non-coding RNA [1]. Meanwhile, Ruvkun’s group revealed that the expression of a nuclear protein encoded by another heterochronic gene, *lin-14*, is negatively regulated by its 3′ untranslated region (UTR) [5]. With their collaboration, Ruvkun’s group showed that lin-4 binds to the complementary sequence of the 3′ UTR of lin-14 mRNA to inhibit its translation [2]. After the discovery of lin-4 and lin-14 crosstalk in *C. elegans*, Ruvkun’s group reported a second miRNA, let-7, in 2000, and its widespread conservation across animals, including humans [6,7]. Their pioneering work altered the central dogma concept, which traditionally states that genomic information flows from DNA to RNA to protein. Instead, they introduced a groundbreaking concept that non-coding RNAs, which do not code for proteins, play a critical role in regulating gene expression at the post-transcriptional level through RNA-RNA interactions.

Since then, miRNAs, which are ~22-nucleotide small non-coding RNAs, have expanded the RNA research field [8,9,10], culminating in the 2024 Nobel Prize in Physiology and Medicine for their groundbreaking discovery. Small RNAs, including miRNAs, secondary siRNAs, and heterochromatic siRNAs, also play important roles in plant biology [11,12,13,14]. With the advent of next-generation sequencing, more than 250 and 1900 miRNA gene annotations have been reported for *C. elegans* and humans, respectively [15]. In mammals, approximately 500 (to 1000) canonical miRNA genes meet the stringent criteria for structure, levels of expression, conservation of sequences, as well as targets. Therefore, miRNAs are the best-defined large family of non-coding RNAs known to date.

As canonical miRNAs, such as lin-4 and let-7, exhibit temporal expression during development in *C. elegans* and control the timing of development, miRNA expression patterns are highly specific to different cell types and tissue types [16,17]. Despite the large number of miRNAs, only a small subset is specifically expressed in each tissue in the mammalian system [16]. This can be explained by the existence of super-enhancers (SEs), where master transcription factors (TFs) bind densely to shape cell type-specific expression patterns of protein-coding and miRNA-coding genes for proper development and homeostasis [16,18]. Moreover, recent studies have unveiled the complexity of miRNA regulatory mechanisms such as miRNA co-targeting; miRNA 3′ tailing and 3′ trimming; and target-directed miRNA degradation (TDMD), which affects miRNA abundance [19,20,21]. These findings emphasize the importance of regulating miRNA levels and their role in fine-tuning gene expression. miRNAs have adapted across species to develop these mechanisms, allowing for varying degrees of gene repression and the precise control of gene regulation. In this review, we summarize the latest insights into miRNA regulatory mechanisms and the diseases that arise from their perturbations, mainly focusing on cancers and neuronal diseases.

## 2. miRNA Biogenesis

### 2.1. Canonical miRNA Biogenesis

**Figure 1:**
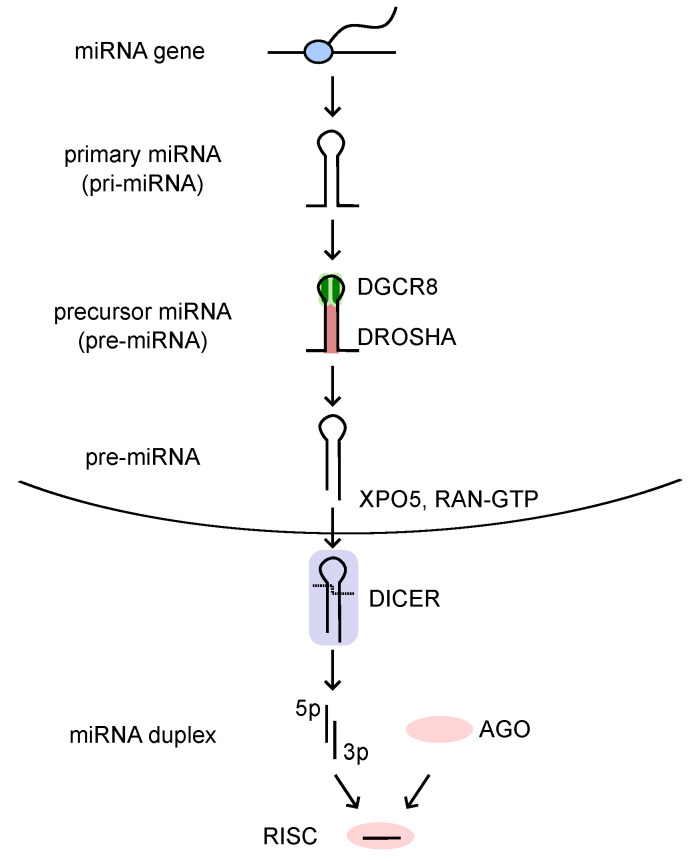
Canonical miRNA biogenesis. miRNA genes are transcribed into primary miRNAs (pri-miRNAs) by RNA polymerase II. Pri-miRNAs are first processed by the DROSHA-DGCR8 microprocessor complex to form pre-miRNAs. These pre-miRNAs are then exported from the nucleus by Exportin-5 (XPO5) and RAN-GTP. Further processing with DICER results in the formation of miRNA duplexes. The guide strand is incorporated into AGO protein to form an RNA-induced silencing complex (RISC) that suppresses gene expression.

The canonical miRNA biogenesis pathway is a stepwise process (Figure 1) [17,20,21]. First, miRNA genes are transcribed into primary miRNAs (pri-miRNAs) by RNA polymerase II. Most miRNA genes reside in either intragenic or intergenic regions [22]. Pri-miRNAs have hairpin-like structures, which possess several important features for mature miRNA generation [23].

Subsequently, the DROSHA-DGCR8 heterotrimer endonuclease complex processes pri-miRNAs, generating precursor miRNAs (pre-miRNAs) [24,25,26]. Pre-miRNAs are exported from the nucleus to the cytoplasm via Exportin-5 (XPO5) and RAN-GTP [27]. In the cytoplasm, pre-miRNAs undergo further processing by DICER endonucleases, forming a miRNA duplex with 5p and 3p strands [28,29,30,31,32]. One strand (called the guide strand) is loaded onto Argonaute (AGO) proteins to form an RNA-induced silencing complex (RISC), while the other strand (called the passenger strand) is eliminated [33]. The choice of the strand is determined by the binding between the 5′ sequence of the miRNA duplex and the MID domain of AGO and it reflects the two parameters of the miRNA duplex: the identity of the 5′-nucleotide and the thermodynamic stability of the two ends of the miRNA duplex. The strand is favored if its 5′-nucleotide is adenine or uracil and its 5′-end is thermodynamically unstable [34,35]. After mature miRNAs are loaded on the AGO proteins, the RISC binds to the complementary sequence in the 3′ UTR of the target mRNAs [1,2,36,37,38]. Together with TNRC6 (GW182) and accessory proteins, miRNAs destabilize target mRNAs and/or inhibit their translation [20].

### 2.2. Mutations in miRNA Biogenesis Pathway and Associated Human Diseases

Abnormalities in miRNA biogenesis pathway are associated with various human diseases (Table 1). These include mutations in *DROSHA*, *DGCR8*, and *DICER1* in cancer, and recently identified *AGO* mutations in neurodevelopmental disorders [39]. *DICER1* mutations are associated with a broad spectrum of hereditary cancer predisposition syndrome (so-called *DICER1* syndrome). The significance of mutations in *DROSHA* and *DICER1* in cancer was extensively reviewed [21,39]. Therefore, in this review, we focus on *DGCR8* and *AGO* mutations and their associations with human diseases.

#### 2.2.1. DGCR8 (DiGeorge Syndrome Critical Region 8)

DGCR8 associates with the RNase III endonuclease DROSHA to form a microprocessor complex. Properly formed microprocessor complexes subsequently cleave pri-miRNAs and produce pre-miRNAs. The *DGCR8* gene is located on chromosome 22q11.2.

The 22q11.2 microdeletion, associated with DiGeorge syndrome, is linked to behavioral and cognitive deficits [44,45,46,47] along with a significantly increased risk of schizophrenia [48]. The estimated prevalence of this condition is ~5 per 10,000 individuals as of 2021 [72]. Currently, the treatment options are limited to symptomatic management. Mice harboring the human-mimic 22q11.2 microdeletion, Df(16)A^+/–^, display impairments in working memory, cognition, and behavior [73]. The brains of Df(16)A^+/−^ mice exhibit a reduced density of dendritic spines and glutamatergic synapses, along with impaired dendritic growth [74]. *Dgcr8* haploinsufficiency alone replicates the phenotypes observed in Df(16)A^+/−^ mice [73]. In *Dgcr8*^+/−^ mice, electrophysiological and morphological analyses have shown that layer 5 pyramidal neurons display short-term synaptic depression and diminished potentiation [75]. Some pri-miRNAs from the prefrontal cortex and hippocampus show elevated expression levels, whereas mature miRNA levels are reduced, indicating disrupted miRNA biogenesis [73,76,77,78] (characterized well in [79]). All studies cited here have demonstrated that miR-185 is downregulated in mouse models. Neuronal differentiation experiments using induced pluripotent stem (iPS) cells derived from two patients with schizophrenia carrying the 22q11.2 microdeletion revealed several abnormalities, including the shrinkage of neurospheres, a reduction in the number of neurons, and defects in neuronal differentiation [77]. miRNA expression profiling showed a decrease in the levels of miR-17~92 cluster and miR-185, which partially aligns with the findings from the disease model mice. These results highlight the critical role of proper miRNA expression levels in brain development. One study specifically focused on miR-185, which is also encoded in the 22q11.2 region [80]. The authors showed that miR-185 was significantly repressed and the expression levels of the target mRNA, Mirta22, were increased in Df(16)A^+/–^ mice. Studies with neurons derived from Df(16)A^+/–^ mice revealed that alterations in miR-185 and Mirta22 are responsible for defects in the spine and dendrite development [80].

*DGCR8* mutations are associated with Wilms tumor [42,43,50], papillary thyroid cancer (PTC) [50], familial multinodular goiter [50], and schwannomatosis [50]. The most frequent and functionally relevant hotspot mutation is the E518K mutation in the double-stranded RNA-binding domain (dsRBD) of DGCR8. Comprehensive bioinformatics analysis of the miRNA and RNA profiles in every case revealed that this mutation perturbs pri-miRNA processing. Collectively, these studies highlight the pivotal role of *DGCR8* as a pri-miRNA processing factor involved in both neuronal development and cancer progression.

#### 2.2.2. AGO (Argonaute)

AGO proteins play crucial roles in the miRNA pathway as the core protein component of RISC. In mammals, there are four AGO paralogs: AGO1, AGO2, AGO3, and AGO4 [81].

*AGO1*, *AGO3*, and *AGO4* are located on chromosome 1p34.3. Microdeletion of 1p34.3 leads to haploinsufficiency of *AGO1* and *AGO3*, which is potentially associated with neurocognitive defects [59]. Five patients with the 1p34.3 microdeletion were shown to display psychomotor developmental delays with several characteristics, including early feeding difficulties, hypotonia, and delays in motor and speech development [59].

Additionally, a recent study identified heterozygous germline mutations in *AGO2* in patients with neurological development disorders (NDDs) [62]. They discovered 13 heterozygous mutations in *AGO2* in 21 patients, all of whom presented with intellectual disability (ID), delayed motor development, and impaired speech and receptive language skills. Approximately 50 to 60% of patients exhibit hypotonia, gait abnormalities, and symptoms of autism spectrum disorder (ASD). Magnetic resonance imaging (MRI) revealed abnormalities in the corpus callosum, confirming the neurological effects of *AGO2* mutations. Although less common, some patients display vision problems, breathing difficulties, craniofacial anomalies, as well as dental, skeletal, and heart defects.

These single amino acid mutations were identified at various positions, but spatially clustered within the AGO2 protein structure at the helix-7/L1 interface; the hinge region of L1; and the loop in the PIWI domain, which recognizes the guide–target duplex [62]. Upon overexpression in *AGO2*-depleted cells, all patient-derived *AGO2* mutants exhibited impaired shRNA-mediated target silencing. While multiple aspects of RISC formation and activity, including interaction with DICER, TNRC6 family, and miRNAs and slicing activities, were not largely affected by most of the mutants, these mutants showed a reduction in phosphorylation signals at the C-terminal PIWI domain and target release. AGO2 phosphorylation is thought to be a crucial step in maintaining the global efficiency of miRNA-mediated silencing [82,83]. A set of highly conserved residues (S824–S834) in the C-terminal PIWI domain is hierarchically phosphorylated by CSNK1A1 upon target mRNA engagement, followed by rapid dephosphorylation by the ANKRD52–PPP6C phosphatase complex [82,83]. Given that this phosphorylation cycle may be important for recycling the RISC from one target mRNA to another, the pathogenic *AGO2* mutations appear to attenuate the global efficiency of the repression of target mRNAs. This was further supported by the molecular dynamics simulations, suggesting an impaired guide–target duplex unwinding function of AGO2 [62]. RNA sequencing (RNA-seq) analysis of patient-derived fibroblasts revealed global transcriptome changes, with commonly altered genes being enriched in mitosis- and cell cycle-regulated pathways in Gene Ontology analysis [62].

Mutations in *AGO1* have also been associated with NDDs, such as ID, ASD with language impairment, and behavioral features [57,58,60,61,63]. In the report by Schalk et al. [63], 15 mutations in *AGO1* were identified from 28 individuals. Although the functional significance of these mutations remains elusive, most *AGO1* mutations are spatially clustered along the sides of the L1 and PAZ domains facing one another and are predicted to affect AGO1 dynamic conformational change [49]. Ambros’s group has recently demonstrated that several *AGO1* mutations affect miRNA function in *C. elegans* models [84]. *C. elegans* harboring mutations in *Alg-1* (*AGO1* orthologue), corresponding to four mutations reported in patients, exhibited increased lethality under stress [84]. Small RNA-seq analysis revealed that both miRNA expression levels and the ratio between the miRNA passenger and guide strands were altered depending on the mutation, leading to mutation-specific perturbed proteome profiles [84]. A detailed investigation of RISC formation and target repression suggested that the mutated *Alg-1* sequestrated miRNAs, thereby eliciting antimorphic effects. Additionally, the genes that were perturbed in these mutants included NDD-related genes.

These pathogenic conditions, characterized by mutations in *AGO1* or *AGO2* and unique phenotypes, are now categorized as Argonaute (AGO) syndrome [62,63]. According to the AGO Alliance website (https://argonautes.ngo/en, accessed on 1 March 2025), approximately 85 patients have been diagnosed with this disease, and this number is expected to increase. Although there is currently no standard treatment for AGO syndrome, Ambros’s group suggested that the pathological effects of NDD-related mutations in *AGO* are associated with disturbances in proteostasis caused by global perturbations in gene expression [84]. These studies lay the foundation for understanding the underlying mechanisms of AGO syndrome, particularly how these mutations impact the brain and neuronal development by impairing miRNA biogenesis and gene repression.

## 3. Diverse Modes of miRNA Targeting

### 3.1. miRNA Target Recognition

**Figure 2:**
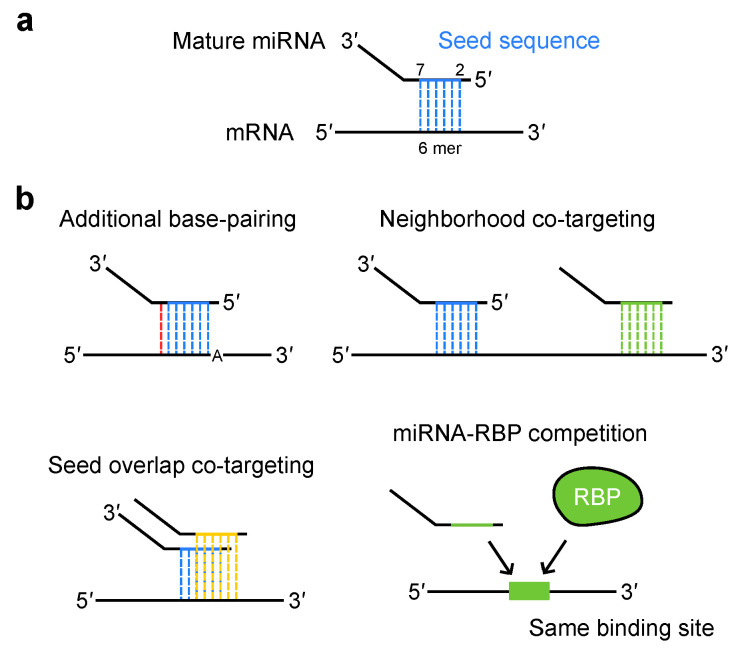
Variability in miRNA targeting. (**a**) The seed sequence of the miRNA (2–7 nucleotide from the 5′-end) pairs with target mRNAs through Watson–Crick base pairing, ensuring specificity in gene regulation. (**b**) miRNA targeting involves diverse mechanisms. RBPs that share the same binding sites with miRNAs are sequestered and lose their activity. RBP: RNA-binding protein.

During target recognition by RISC, typically, the seed sequence of miRNAs, spanning the second to seventh nucleotides from the 5′-end, typically pairs with target mRNAs through Watson–Crick base pairing (Figure 2a) [17]. This 6 mer seed match is augmented by either an additional match to the eighth nucleotide (7 mer-m8) or an A at the first nucleotide (7 mer-A1): the coexistence of both further potentiate target repression (8 mer) (Figure 2b) [85,86]. Additional pairing to the 3′ region of the miRNA complements the target recognition. In most cases, the extent of gene repression by miRNAs is not a result of a single miRNA or target site pair acting alone. Because the seed sequence is composed of only six or seven nucleotides, individual miRNAs can target hundreds of different mRNAs. Moreover, the 3′ UTR of mRNAs have multiple miRNA target sites. Hence, multiple miRNAs and their target sites cooperate to achieve robust repression and form complex regulatory networks.

### 3.2. miRNA Co-Targeting

In a combinatorial mode of miRNA targeting, both identical and different miRNAs are known to simultaneously target neighboring sites located in close proximity and achieve synergistic repression [85,87,88]. This mechanism, known as “neighborhood co-targeting”, involves multivalent interactions between TNRC6 and multiple AGO proteins [87]. Another miRNA co-targeting scenario is “seed overlap co-targeting”, in which different miRNAs with similar sequences target overlapping regions of the mRNA. Although these miRNAs cannot bind to the same region at the same time, their combined action leads to additive repression and increased susceptibility to downregulation by two miRNAs [89]. These co-targeting modes appear to be prevalent in both conserved miRNAs and target sites. The highly conserved target sites of broadly conserved miRNAs can be categorized into two groups: those conserved from humans to *Coelacanth*, and those that were recently acquired among eutherian mammals [89]. The former has a stronger association with both “neighborhood” and “seed overlap” miRNA co-targeting [89]. This suggests that evolutionarily important genes are regulated by diverse mechanisms to maintain their expression. Impaired miRNA co-targeting was implicated in human diseases, such as systemic lupus erythematosus (SLE). In a mouse model of SLE, the expression levels of miR-128 and miR-148a are reduced [89]. Since these miRNAs share similar sequences, their co-targeting gene, *KLF4*, is upregulated. This upregulation contributes to the production of SLE-associated cytokines. Given that multiple related miRNAs from polycistronic miRNA genes coordinately regulate target genes in the related pathways [90], these modes of co-targeting contribute to coordinated regulation of the biologically related pathways.

In addition to miRNAs, RNA-binding proteins (RBPs) interact with mRNAs and serve as key regulators of gene expression [91,92]. When RBPs and miRNAs recognize the same sequence motifs, miRNAs can sequester the RBPs, leading to the modulation of RBP activity (see Section 4.2). In summary, the seed sequences of miRNAs employ multiple targeting mechanisms to ensure precise gene repression.

## 4. Super-Enhancers (SEs) Drive Cell Type-Specific miRNA Expression

### 4.1. Super-Enhancers (SEs) and miRNAs

**Figure 3:**
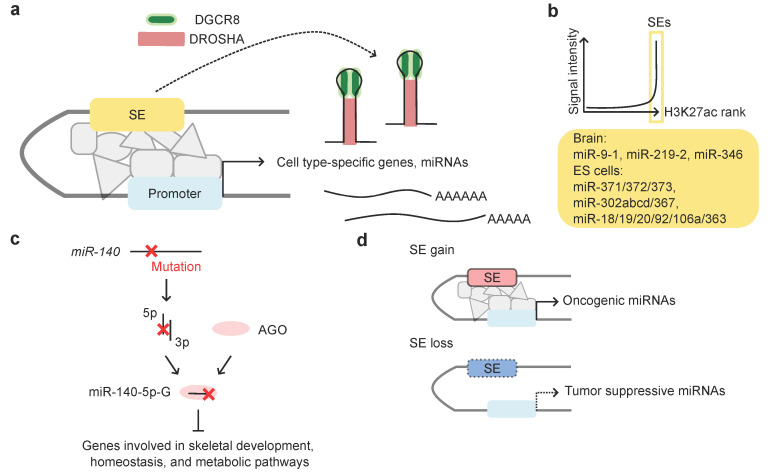
SEs regulate master miRNA expression. (**a**) SEs regulate the expression of key TFs and master miRNAs, thereby shaping cell identity. SE: super-enhancer; gray objects: proteins accumulated around the SE. (**b**) SEs are characterized by H3K27ac enrichment in ChIP-seq analysis. Examples of SE-associated miRNAs are presented in [16]. (**c**) Mutations in *miR-140* alter miRNA strand selection, favoring the mutation-incorporated miR-140-5p strand (miR-140-5p-G), leading to aberrant skeletal development via GOF effects. (**d**) In cancer, gain and loss of SEs are associated with oncogenic and tumor suppressive miRNAs, respectively.

All cells of an organism possess the same genome. However, each differentiated cell is programmed to express a specific set of genes unique to that cell type [93]. This is also the case for miRNAs, as only a small subset of miRNAs is specifically expressed in each mammalian tissue [16]. The regulation of gene expression is primarily orchestrated by TFs. Super-enhancers (SEs), which are densely bound by master TFs and are present in numbers of 200–800 in each cell type, define cell type specificity by driving the robust expression of key identity genes [18]. A fraction of SEs is located in proximity to key miRNA genes, the expression of which are highly abundant in the corresponding cell type [16]. In human embryonic stem cells (hESCs), SE-driven miRNAs participate in the regulatory circuit along with OCT4, SOX2, and NANOG, which collectively regulate pluripotency gene expression [94,95,96]. SEs were suggested to induce pri-miRNA transcription of key miRNA genes and promote pri-miRNA processing. DGCR8 accumulates at SEs and facilitates the recruitment of DROSHA, thereby inducing cooperative and efficient pri-miRNA processing (Figure 3a) [16]. To ensure tissue identity, genes co-expressed with miRNAs are thought to have evolved to selectively avoid target sites matching the miRNAs. Consistent with this, genes co-expressed with SE-associated miRNAs show strong depletion of target sites [16]. This implies that SE-driven gene expression was optimized through the co-evolution of genes and miRNAs.

miRNAs such as miR-9, miR-124, miR-128a/b, miR-219, and miR-346 are predominantly expressed in the brain, highlighting their critical roles in neural development and function [16,97]. Among these, miR-9, miR-219, and miR-346 were identified as SE-associated miRNAs based on their H3K27ac enrichment profiles (Figure 3b) [16].

### 4.2. SE-Associated miRNAs and Human Diseases

Mutations in miRNA seed sequences are linked to several human diseases, including non-syndromic progressive hearing loss associated with *miR-96* seed mutations [98,99], endothelial dystrophy, iris hypoplasia, congenital cataract, and stromal thinning (EDICT) syndrome associated with *miR-184* mutations [100,101,102], ocular coloboma associated with *miR-204* mutations [103], and skeletal dysplasia associated with *miR-140* mutations (Table 2) [91]. These seed mutations can affect miRNA abundance and gene repression levels, leading to diverse outcomes. For instance, in patients with ocular coloboma harboring *miR-204* mutations, the miR-204-regulated apoptotic pathway is activated, causing cone and rod cell death [103]. Besides miRNA gene mutations, germline hemizygous deletions of the miRNA-17~92 cluster were identified in patients with Feingold syndrome [104,105].

Mutations in SE-associated miRNAs were identified in patients with skeletal dysplasia [91]. The skeletal dysplasia (spondyloepiphyseal dysplasia (SED) MIR140 type Nishimura, OMIM #618618) is characterized by a disproportionately short stature and spondylar and epiphyseal abnormalities. The seed sequence of chondrocyte-specific SE-associated *miR-140* was found to be mutated in these patients. The second nucleotide from the 5′-end of the miR-140-5p strand was altered from A to G. Although the pre-miRNA of miR-140 yielded both 5p and 3p strands, with a preference for the 3p arm, small RNA-seq analysis revealed an increase in miR-140-5p-G levels, consistent with alterations in miRNA strand selection. miR-140-5p-G suppresses de novo target genes involved in skeletal development and metabolic pathways, which are distinct from the target genes of the wild-type miR-140 species (Figure 3c). The mutant miR-140-5p seed competes with the conserved RNA-binding protein Ybx1 for the overlapping binding sites of these de novo target genes, achieving potent repression of otherwise weak targets that are not supported by target site evolution. Importantly, *miR-140* A>G mutant mice show distinct phenotypes compared to *miR-140* null mice. Both mice exhibit short stature and nasal bone abnormalities; however, delayed secondary ossification is unique to mutant mice, indicating gain-of-function (GOF) phenotypes.

Alterations in SE-associated miRNAs are frequently observed in cancer. An analysis of chromatin immunoprecipitation (ChIP)-sequencing (ChIP-seq) in cancer cells and The Cancer Genome Atlas (TCGA) database revealed widespread alterations in SE distribution near the oncogenic and tumor suppressive miRNA genes [16]. SEs have gained close proximity to oncogenic miRNAs, whereas they have often lost close proximity to tumor-suppressive miRNAs (Figure 3d). Additionally, high expression levels of SE-gained oncogenic miRNAs are associated with a poor prognosis, suggesting their potential as prognostic biomarkers.

In small cell lung cancer (SCLC), four molecular subtypes were classified based on the expression of master transcriptional regulators including ASCL1, NEUROD1, POU2F3, and YAP1. In the ASCL1 subtype, ASCL1 positively regulates SE-associated miRNAs such as miR-7, miR-375, miR-200b-3p, and miR-429, while negatively regulating miR-455-3p [106]. Of note, miR-455-3p is not negatively regulated in other SCLC subtypes, suggesting that ASCL1-mediated miRNA expression profiles can provide valuable insights into the subtype-specific mechanisms of cancer development.

## 5. The Post-Transcription Regulation of miRNA Expression: 3′ Tailing, 3′ Trimming, and TDMD

**Figure 4:**
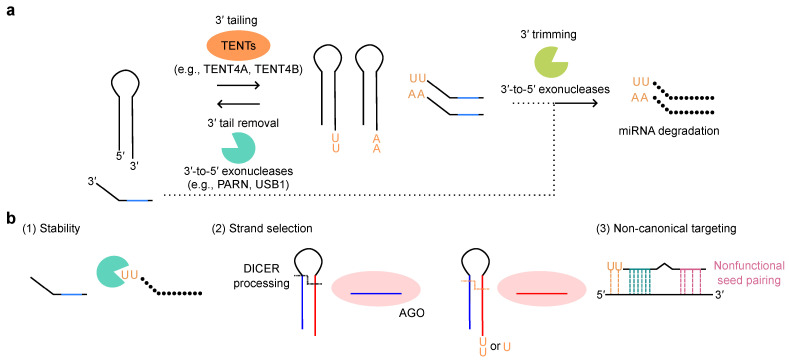
miRNA 3′ tailing and 3′ trimming. (**a**) Overall mechanism of miRNA 3′ tailing and 3′ trimming. TENTs: terminal nucleotidyl transferases. (**b**) 3′ tailing and 3′ trimming regulate miRNA stability, strand selection, and targeting.

Advancements in technologies such as next-generation sequencing and mass spectrometry have revealed the presence of post-transcriptional modifications in miRNAs [97,107,108,109]. These include base modifications, such A-to-I editing, 3′-end tailing, and 3′ trimming (Figure 4a) [97,107,110], and epitranscriptomic modifications such as m^6^A, m^5^C, m^7^G, and o^8^G [111,112].

### 5.1. miRNA 3′ Tailing and 3′ Trimming: Post-Transcriptional Modifications of miRNAs

The addition of bases at the 3′-end (3′ tailing) is catalyzed by several terminal nucleotidyl transferases (TENTs), such as TUT4/ZCCHC11, TUT7/ZCCHC6, TENT2/PAPD4/GLD2, TENT4B/PAPD5/GLD4, TENT4A/PAPD7, TUT1/Star-PAP/RBM21, and MTPAP/PAPD1 [19,113,114,115]. The 3′-end nucleotide tails added to miRNAs can recruit certain processive miRNA degraders, such as DIS3L and DIS3L2, leading to their 3′-to-5′ degradation (3′ trimming (shortening or decay)) (Figure 4a). Moreover, 3′ tailing and 3′ trimming is frequently triggered by extensive complementarity between miRNA and target mRNA [116]. Furthermore, the heterogeneity of 3′-end can be controlled by the balance of 3′ tailing and the reverse process: the 3′-end extension can be removed by deadenylase activities of the 3′-to-5′ exonucleases, PARN and USB1 (Figure 4a) [68]. Consequently, these mechanisms affect miRNA abundance (Figure 4a). For 3′ tailing and 3′ trimming, several RBPs were implicated in recruiting and/or cooperating with 3′-end modifiers. For example, Lin28 recruits TUT4/ZCCHC11 or TUT7/ZCCHC6 to let-7 [117,118,119,120,121,122], CUGBP1 recruits PARN to miR-122 [123], and QKI-7 recruits TENT2/PAPD4/GLD2 to miR-122 [124]. In this subsection, we focus on the physiological roles of 3′ tailing and 3′ trimming of miRNAs, which are crucial for miRNA stability, strand selection, and function (Figure 4b).

Additionally, 3′-end uridylation and adenylation have been extensively studied for their roles in regulating miRNA stability. In mouse embryonic stem cells (mESCs) and certain cancer cells, TUT4/ZCCHC11 or TUT7/ZCCHC6 catalyze oligo-uridylation of pre-let-7, leading to its destabilization [117,118,119,120,121,122]. An in vitro DICER processing assay revealed that oligo-uridylation prevents pre-let-7 from being processed. The knockdown of *TUT4/ZCCHC11* or *TUT7/ZCCHC6* increases the levels of pre-let-7 and mature let-7 miRNAs, promoting the repression of the target genes. Further analysis of this mechanism has shown that oligo-uridylated pre-let-7 is degraded by the 3′-to-5′ exonuclease DIS3L2 [125]. While oligo-uridylation induces the degradation of pre-let7, mono-uridylation of pre-let-7 with a shorter (1 nucleotide) 3′ overhang promotes DICER-mediated processing, highlighting the functional duality of pre-miRNA uridylation [126]. During the mono-uridylation step, an additional enzyme, TENT2/PAPD4/GLD2, functions redundantly with TUT4/ZCCHC11 and TUT7/ZCCHC6 [126]. Moreover, the uridylation of miRNAs can influence their function. For instance, miR-26 loses the ability to silence its target, IL-6 mRNA, when uridylated by TUT4/ZCCHC11 [127].

Beyond uridylation, TENT2/PAPD4/GLD2 was characterized as a poly(A) polymerase. TENT2/PAPD4/GLD2 mono- or oligo-adenylates the 3′-end of miR-122, leading to its stabilization in both human and mouse liver cells [128]. miR-122 is highly expressed in the liver and plays a crucial role in liver development. Mass spectrometry and in vitro DICER processing assays have revealed that adenosine is added to the 3′-end of miR-122. Deadenylation of miR-122 by PARN induces its degradation, further supporting the protective role of miR-122 adenylation [123]. In contrast, studies on the oncogenic miRNA, miR-21, have shown the destabilizing effect of miRNA adenylation, with TENT4B/PAPD5/GLD4-mediated mono-adenylation promoting its degradation by PARN [129]. Furthermore, a comprehensive analysis of miRNAs expressed in human THP-1 monocytic cells has indicated that, in most cases, miRNA 3′-end adenylation appears to interfere with the uptake to AGO proteins, without affecting stability [110]. Taken together, these findings indicate that 3′-end adenylation exerts cell- and miRNA-specific effects on the stability of multiple miRNAs.

In addition, 3′ tailing can influence miRNA strand selection [130] and miRNA target preferences [131]. TUT4/ZCCHC11- or TUT7/ZCCHC6-mediated pre-miR-324 uridylation alters its conformation, DICER processing, strand choice, and choice of mRNA targets. Uridylated pre-miR-324 favors 3p strand selection, enhancing proliferation, whereas, unmodified pre-miR-324 favors 5p strand selection, leading to the repression of proliferation [130]. In addition, 3′ uridylation enables base pairing with adenosine residues in non-canonical target mRNAs, resulting in an expanded targeting mechanism termed tail-U-mediated repression (TUMR) [131]. This suggests that miRNAs can recognize and regulate non-seed matched non-canonical targets when subjected to 3′ tailing.

Collectively, these findings highlight the complex patterns of miRNA post-transcriptional modifications and their various effects on miRNA functions. A more detailed investigation of each molecular mechanism is required to fully characterize the physiological roles of miRNA 3′ trimming and 3′ tailing.

### 5.2. Target-Directed miRNA Degradation (TDMD)

TDMD is a novel mechanism that is associated with miRNA 3′ processing and regulates miRNA expression levels [116,132,133,134,135]. Some miRNAs bind to target mRNAs with extensive base pairing, involving not only the 5′ seed sequence, but also the 3′-end, with mismatches in the central region. This extensive base pairing induces ZSWIM8-mediated polyubiquitination on AGO proteins, which are subsequently degraded in a proteasome-dependent manner, finally leading to miRNA degradation [113,114]. Both 3′ tailing and 3′ trimming of miRNAs loaded onto AGO were previously considered crucial steps in miRNA regulation [116,136,137]. However, recent studies have suggested that TDMD, 3′ trimming, and 3′ tailing are independent mechanisms [133,134,138]. In *ZSWIM8*-knockout cells, 3′ tailed and 3′ trimmed miRNAs accumulated [133,134]. Using the artificial miRNAs with 2′ -O-methyl group at the 3′-end, which prevents 3′ tailing and 3′ trimming, these reports demonstrated that these miRNAs could still undergo degradation in the presence of ZSWIM8 [133,134]. These findings suggest that 3′ trimming and 3′ tailing pathway and ZSWIM8-mediated TDMD pathway are separable. In addition, PARN and DIS3L2 appeared to be dispensable for extensive 3′ tailing and 3′ trimming in *ZSWIM8*-knockout cells [133].

Mechanisms underlying TDMD remain an active area of investigation. Unresolved questions remain regarding the regulatory mechanisms involving unidentified deubiquitinating enzymes and substrate preferences. In addition, TDMD was suggested to be more efficient in neuronal cells than in other tissues [139,140]. Understanding these mechanisms will help to clarify the possible involvement of TDMD in pathological conditions.

### 5.3. miRNA 3′ Tailing and 3′ Trimming in Human Diseases

Several studies have reported alterations in miRNA 3′ tailing in cancer cells. An analysis on TCGA data revealed that adenylated miR-21 levels and their degradation rates are low in many cancer types [129]. Consequently, the expression of miR-21 targets, such as p53, is strongly repressed. Additionally, human papilloma virus (HPV)-induced cervical cancer is associated with the downregulation of TUT7/ZCCHC6 and the upregulation of TENT2/PAPD4/GLD2 and TENT4A/PAPD7, although the impact of these changes on miRNA 3′ tailing remains to be determined [141].

**Figure 5:**
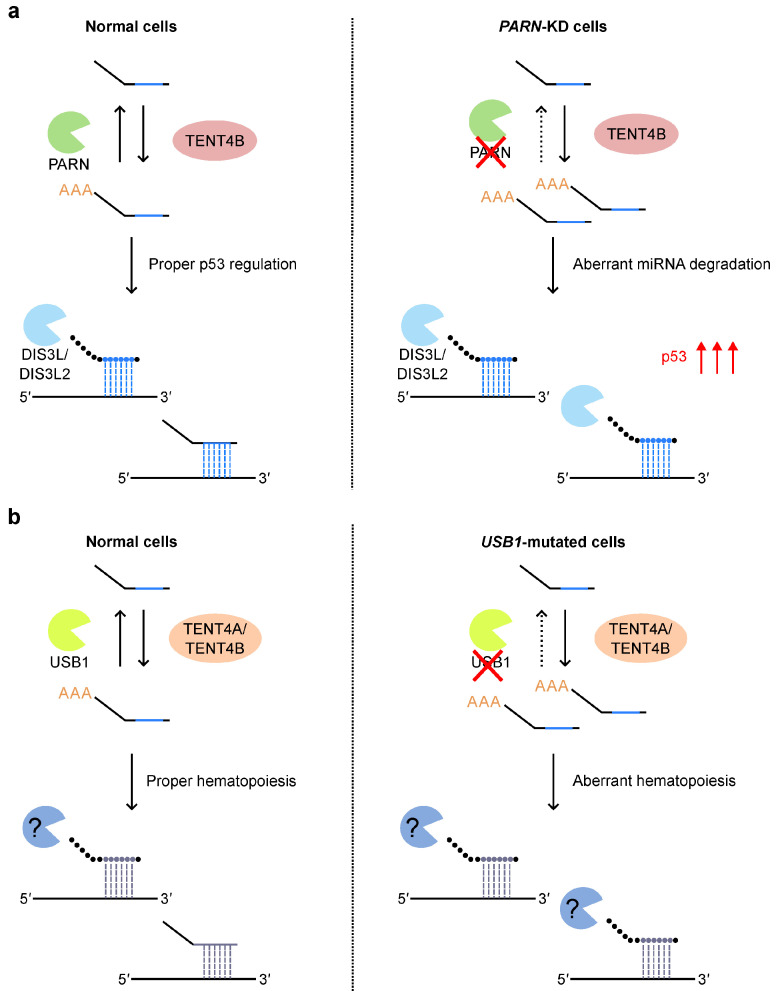
The roles of PARN and USB1 in human diseases. (**a**) In *PARN* wild-type cells, PARN and TENT4B/PAPD5/GLD4 fine-tune miRNA 3′-end adenylation, working alongside DIS3L2-mediated miRNA degradation to ensure normal p53 regulation. However, in *PARN*-knockdown (KD) cells, similar to patients with DC, enhanced DIS3L2-mediated miRNA degradation leads to p53 upregulation. (**b**) In *USB1* wild-type cells, USB1, TENT4A/PAPD7, and TENT4B/PAPD5/GLD4 fine-tune miRNA 3′-end adenylation, working with miRNA degradation to ensure normal hematopoiesis. However, in *USB1*-mutated cells, similar to patients with PN, enhanced miRNA degradation leads to aberrant hematopoiesis.

In addition to its involvement in cancer, mutations in the 3′-end processing enzymes, PARN and USB1, were implicated in the human diseases, dyskeratosis congenita (DC) and poikiloderma with neutropenia (PN), respectively (Table 1) [67,68,71]. DC is characterized by abnormal telomere length. Patients present with three major symptoms: nail atrophy, oral leukoplakia, and cutaneous hyperpigmentation. PARN was reported to be a regulator of the stability of certain types of ncRNAs, including human telomerase RNA (hTR), in fact, it is evident that telomere length is affected in DC patients [64,65,66]. However, it remains unclear whether the patient phenotypes can be fully explained by the telomere length regulation. Recently, it was discovered that PARN deadenylates TENT4B/PAPD5/GLD4-adenylated miRNAs in normal cells to protect against DIS3L2- and DIS3L-mediated miRNA degradation (Figure 5a) [67]. Thus, the four enzymes, PARN, TENT4B/PAPD5/GLD4, DIS3L, and DIS3L2, collaborate to precisely regulate miRNA expression. In *PARN*-knockdown cells, miRNAs involved in regulating p53 expression, such as miR-25, miR-92, miR-214, miR-331, miR-380, miR-1285, and miR-3126, are downregulated, leading to p53 upregulation [67]. This cooperative p53 upregulation aligns with a previously reported increase in p53 activity in patient-derived DC cells harboring *PARN* mutations [64]. Taken together, these results suggest that PARN depletion alters miRNA profiles, leading to a global increase in p53 activity, which may underlie the pathology of DC.

Mutations in *USB1* (also known as *C16orf57*) are linked to PN [69,70]. PN is characterized by poikiloderma, a skin condition involving an erythematous rash, with a decrease in neutrophils and a predisposition to myelodysplastic syndrome (MDS) and acute myeloid leukemia (AML). USB1 is traditionally thought to function in U6-snRNA processing; however, its physiological role in PN remains unclear. In 2023, Jeong et al. reported the involvement of USB1 in hematopoietic development using CRISPR-Cas9-engineered *USB1*-mutant hESCs (Figure 5b) [71]. The mutations frequently observed in patients did not affect splicing, which is consistent with a previous report [142]. In contrast, USB1 is involved in the regulation of miRNA expression during hematopoietic development. In *USB1*-mutated undifferentiated cells, 82 of the 374 miRNAs are altered. Similarly, in *USB1*-mutated hematopoietic progenitor cells, 131 of the 771 miRNAs exhibit changes in expression levels. Kyoto Encyclopedia of Genes and Genomes (KEGG) analysis indicates that these differentially expressed miRNAs are involved in cancer progression, suggesting a potential link to oncogenic pathways during hematopoietic differentiation. USB1 deadenylates miRNA substrates both in vivo and in vitro. Genetic or chemical inhibition of TENT4A/PAPD7 and TENT4B/PAPD5/GLD4 rescue the phenotype of *USB1*-mutated cells. These data suggest that TENT4A/PAPD7, TENT4B/PAPD5/GLD4, and USB1 together control the expression of a subset of miRNAs and regulate hematopoietic development.

## 6. The Evolution of miRNA Genes and Its Potential Impacts

Recent studies have shown that miRNA-mediated post-transcriptional regulation is shaped by various factors, such as stepwise biogenesis pathways, seed variability, co-targeting, RBPs, SEs, and post-transcriptional modifications [19,20,21]. These mechanisms introduce additional layers into miRNA-mediated gene regulation, providing clues for understanding when and to what extent miRNAs contribute to optimal regulatory outcomes. As described in Section 3, mutations in the miRNA biogenesis pathway often lead to neuronal phenotypes in human diseases, as exemplified by *DGCR8* and *AGO* involvement. However, the reason why miRNA perturbations predominantly affect the neuronal system remains unclear. This may be partly associated with the correlations between miRNA gene evolution and organismal complexity and unique features of miRNA regulation in the neuronal system [143].

The miRNA biogenesis pathway and the role of miRNAs as post-transcriptional repressors are well conserved from plants to animals. In animals, miRNA genes have evolved in association with metazoan evolution over time [143,144,145]. With the emergence of humans, the brain became larger, featuring an expanded cerebral cortex with approximately 86 billion neurons [144]. Human miRNAs are estimated to be twice as numerous as mouse miRNAs [143]. A comparison of human and chimpanzee brains previously identified multiple species-specific miRNAs [146]. Evolutionarily new miRNAs are abundant in the human brain, suggesting that miRNA evolution is an important contributor to cognitive and developmental complexity [146]. In association with these findings, previous studies have suggested that miRNAs and target regulation are modulated in a more complicated manner in brain tissue compared to other tissues [88,147]. First, a set of neuronal miRNAs regulates gene expression through “neighborhood” co-targeting to achieve robust repression [88,147]. For instance, SE-associated miR-9 and typical enhancer (TE)-associated miR-124 or TE-associated miR-137 and miR-138 exhibit spatiotemporal co-expression in the brain, enabling combinational gene regulation [88]. Notably, co-targeting miRNAs vary across developmental stages, as shown in studies on the mouse cerebral cortex (at E14, E17, and P0), indicating their dynamic roles in brain development [147]. Therefore, despite their low expression levels, evolutionarily new miRNAs may regulate gene expression in combination with conserved or young miRNAs.

Further, miRNA-mediated gene regulation is modulated by the regulation of the 3′ UTR lengths of mRNAs. In the nervous system, mRNA isoforms with longer 3′ UTRs are highly expressed, offering an increased number of miRNA target sites for miRNA-mediated repression [148]. In neurons, the number of target sites of SE-associated miR-9 and TE-associated miR-137 and miR-124 increase, enabling these miRNAs to regulate their target mRNAs in a brain-specific manner. Moreover, a recent study demonstrated that increased 3′-end trimming is observed during mouse postnatal brain maturation (at P22, P40, P60, and P120) [149]. Analysis of human frontal gyrus samples from individuals aged 2 days to 61 years also showed that approximately 25% of miRNAs exhibit 3′-end shortening with age [149]. This may be associated with faster miRNA turnover in the brain than in other tissues [150].

**Figure 6:**
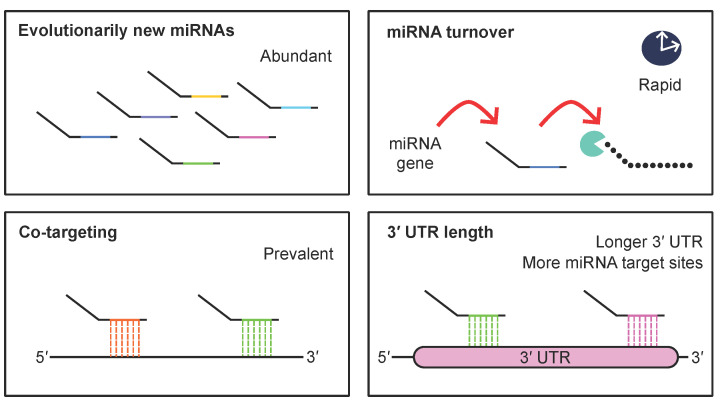
Unique features of miRNA regulation in the brain. In the brain, the miRNA targeting and regulatory processes exhibit unique characteristics. UTR: untranslated region.

As we reviewed in the context of *DGCR8* and *AGO* mutations, NDDs are often associated with impairment of the fine regulation of miRNA abundance and targeting mechanisms. Recent studies have suggested the involvement of miRNAs in bipolar disorder, depression, schizophrenia, and Parkinson’s disease [151,152]. In summary, these findings suggest the unique contribution of miRNAs to gene regulation and disease pathogenesis in the nervous system (Figure 6).

## 7. Conclusions and Perspectives

After three decades of intensive basic research, miRNA studies have transitioned toward clinical applications. miRNAs are now recognized as promising biomarkers for diseases and as potential therapeutic modalities [144,153,154,155]. Major challenges in miRNA-based therapeutics include the development of efficient delivery systems and the minimization of off-target effects. To overcome these obstacles, new modalities such as ligand-mediated conjugation and nanoparticle-based delivery systems were developed [153,155]. However, due to the short length of seed sequences, it remains difficult to accurately predict the functional outcomes of synthetic miRNAs. Recent advances in artificial intelligence (AI) may improve comprehensive research on miRNA targets. Further advances in bioinformatics and experimental research on individual miRNAs will improve therapeutic development.

Despite these significant advancements, several questions remain unanswered. For example, the concept of SEs and their associated miRNAs is relatively novel. The interplay between SE-associated and TE-associated miRNAs and human diseases is not yet fully understood. The balance between SE- and TE-associated miRNAs may vary across diseases, particularly in neuronal disorders. Exploring these interactions will shed light on novel regulatory networks and provide new perspectives on disease mechanisms.

Although not covered in detail in this review, an additional perspective involves miRNA-mediated translation activation [156,157,158,159,160,161,162,163,164]. The target mRNAs described thus far include mRNAs harboring AU-rich elements in their 3′ UTR (e.g., TNFα mRNA), 3′ poly(A)-less mRNA, hepatitis C virus RNA, and mitochondrial mRNA. Despite their potential importance, the mechanisms by which miRNAs evoke translational activation remain largely unknown.

In summary, the ongoing integration of miRNA research with cutting-edge technologies and the exploration of novel mechanisms highlights the dynamics of miRNA-mediated gene regulation. These advancements pave the way for miRNAs to play crucial roles in both fundamental biology and clinical applications.

## Figures and Tables

**Table 1:** Diseases associated with alterations in miRNA regulation.

	Gene	Disease	Phenotypes	References
miRNA biogenesis pathway	*DROSHA*	Wilms tumor (point mutation)	Genitourinary tract cancer	[40,41,42,43]
	*DGCR* *8*	DiGeorge syndrome (22q11.2 deletion)	Congenital heart defects, developmental delay, behavioral and cognitive deficits, learning disabilities, immunodeficiency, hypocalcemia, characteristic facial features, and schizophrenia	[44,45,46,47,48,49]
		Wilms tumor (point mutation)	Genitourinary tract cancer	[42,43]
		Tumor susceptibility syndrome (point mutation, loss-of-heterozygosity)	Euthyroid multinodular goiter (MNG) with schwannomatosis	[50]
	*DICER1*	*DICER1* Syndrome (point mutation, loss-of-heterozygosity)	Pleuropulmonary blastoma, ovarian Sertoli–Leydig cell tumor, cystic nephroma, embryonal rhabdomyosarcoma, and multinodular goiter	[51,52,53,54,55]
		Wilms tumor (point mutation)	Genitourinary tract cancer	[40,41,56]
	*AGO1* *AGO2*	*AGO* syndrome (1p34.3 deletion, point mutation)	Psychomotor developmental delays, neurological development disorder (NDD), intellectual disability (ID), delayed motor development, impaired speech and receptive language skills, and autism spectrum disorder (ASD)	[57,58,59,60,61,62,63]
miRNA 3′-end processing	*PARN*	Dyskeratosis congenita (DC) (point mutation, deletion)	Telomere shortening, nail atrophy, oral leukoplakia, cutaneous hyperpigmentation, pulmonary fibrosis, bone marrow failure, and cancer	[64,65,66,67,68]
	*USB1*	Poikiloderma with neutropenia (PN) (point mutation, deletion)	Poikiloderma, decrease in neutrophils, bone marrow failure, and cancer	[68,69,70,71]

**Table 2:** Mutations in miRNA genes in human diseases.

miRNA Gene	Disease	Phenotypes	References
*miR-96*	Non-syndromic hearing impairment (point mutation)	Progressive hearing loss	[98,99]
*miR-184*	EDICT syndrome (point mutation)	Endothelial dystrophy, iris hypoplasia, congenital cataract, and stromal thinning	[100,101,102]
*miR-204*	Ocular coloboma (point mutation)	Retinal dystrophy	[103]
*miR-140*	Skeletal dysplasia (spondyloepiphyseal dysplasia (SED) MIR140 type Nishimura) (point mutation, SE-associated)	Disproportionately short stature, and spondylar and epiphyseal abnormalities	[91]
*MIR17HG*	Feingold syndrome (13q31.3 deletion)	Microcephaly, short stature, and digital abnormalities	[104,105]

## Abbreviations

The following abbreviations are used in this manuscript:

miRNA	microRNA
UTR	Untranslated region
SE	Super-enhancer
TF	Transcription factor
TDMD	Target-directed miRNA degradation
pri-miRNA	Primary miRNA
pre-miRNA	Precursor miRNA
XPO5	Exportin-5
AGO	Argonaute
RISC	RNA-induced silencing complex
NDD	Neurological development disorder
ID	Intellectual disability
ASD	Autism spectrum disorder
DC	Dyskeratosis congenita
PN	Poikiloderma with neutropenia
RNA-seq	RNA sequencing
SLE	Systemic lupus erythematosus
RBP	RNA-binding protein
ESC	Embryonic stem cell
GOF	Gain-of-function
ChIP-seq	Chromatin immunoprecipitation-sequencing
TCGA	The Cancer Genome Atlas
SCLC	Small cell lung cancer
TENT	Terminal nucleotidyl transferase
TE	Typical enhancer
